# The Effect of Ovariectomy and Orchiectomy on Orthodontic Tooth Movement and Root Resorption in Wistar Rats

**Published:** 2015-12

**Authors:** Massoud Seifi, Baharak Ezzati, Sara Saedi, Mehdi Hedayati

**Affiliations:** a Dept. of Orthodontics, School of Dentistry, Shahid Beheshti University of Medical Sciences, Tehran, Iran.; b Dept. of Prosthodontics, School of Dentistry, Tabriz University of Medical Sciences, Tabriz, Iran.; c Researcher, Shahid Beheshti University of Medical Sciences, Tehran, Iran.; d Research Institute for Endocrine Science, Shahid Beheshti University of Medical Sciences, Tehran, Iran.

**Keywords:** Orthodontic Tooth Movement, Root Resorption, Testosterone, Estrogen, Progesterone

## Abstract

**Statement of the Problem:**

Root resorption (RR) after orthodontic tooth movement (OTM) is known as a multifactorial complication of orthodontic treatments. Hormonal deficiencies and their effect on bone turnover are reported to have influences on the rate of tooth movement and root resorption.

**Purpose:**

This study was designed to evaluate the effect of female and male steroid sex hormones on tooth movement and root resorption.

**Materials and Method:**

Orthodontic appliances were placed on the right maxillary first molars of 10 ovariectomized female and 10 orchiectomized male Wistar rats as experimental groups and 10 female and 10 male healthy Wistar rats as control groups. NiTi closed-coil springs (9mm, Medium, 011"×.030", Ortho Technology^®^; Tampa, Florida) were placed between the right incisors and the first right maxillary molars to induce tipping movement in the first molars with the application of a 60g force. After 21 days, the rats were sacrificed and tooth movement was measured by using a digital caliper (Guanglu, China). Orthodontic induced root resorption (OIRR) was assessed by histomorphometric analysis after hematoxylin and eosin staining of sections of the mesial root.

**Results:**

The rate of tooth movement was significantly higher in all female rats, with the root resorption being lower in the experimental group. The rate of tooth movement in experimental male rats was significantly higher than the control group (*p*= 0.001) and the rate of root resorption was significantly lower in the experimental group (*p*= 0.001).

**Conclusion:**

It seems that alterations in plasma levels of estrogen, progesterone, and testosterone hormones can influence the rate of OTM and RR. The acceleration in tooth movement increased OTM and decreased RR.

## Introduction

Orthodontic tooth movement (OTM), as an essential component of orthodontic treatment, is achieved by bone remodeling during force application. Orthodontic-induced root resorption (OIRR) is an undesirable pathological consequence of orthodontic treatments, believed to be unavoidable, as in histological studies occurrence of greater than 90% of orthodontically induced inflammatory root resorption has been reported.[1-4] OIRR is usually asymptomatic and minimal with no clinical importance; however in rare cases, severe root resorption (more than ¼ of the root length) ends in root shortening and weakening of the tooth structure.[3, 5-6]

During orthodontic force application, tooth movement occurs as a result of alveolar bone resorption due to the osteoclastic activity on the pressure side and bone deposition due to the osteoblastic induction on the tension side.[6-7] The periodontal ligament cells on the pressure side go through a necrosis process and after the hyaline zone formation, the tooth movement stops. The imbalance between osteoblastic and osteoclastic activities on the pressure and tension sides may result in resorptive areas on the root.[3, 6, 8] During the elimination process of the hyaline zone, mononuclear macrophages and multinuclear gigantic cells may damage the outer layer of the root which consists of cementoblasts. In more severe cases, the dentin may be affected as well. The resorption in dentin is irreversible and is considered a severe damage to the tooth structure.[5-6]

The risk of OIRR is affected by many factors related to the patient and the orthodontic treatment. From all patient-related factors, previous studies demonstrated that systemic factors, including hormone deficiencies and alveolar bone density, could influence OIRR.[1, 3-4,6] The rate of OTM is also affected by many factors including bone turnover.[9] Sex hormones like many other hormones have an impact on bone turnover. The influences of estrogen, progesterone, and testosterone on bone metabolism have been reported in many studies.[7, 10-11] The osteoporosis induced by estrogen and progesterone deficiencies in post-menopausal period results in bone loss and increased bone turnover.[12] Estrogens and androgens have been traditionally known as regulators of bone turnover in women and men.[8] The osteoporosis followed by the estrogen and progesterone withdrawal, affects the alveolar bone, promotes periodontal diseases and is a main cause of tooth loss. In orthodontic treatments, the increased bone turnover induced by lack of these hormones, results in progression of the tooth movement in an unstable pattern.[9, 11, 13] Osteoporosis and loss of bone mineral density caused by aging in male patients have also become an issue recently.[14-15] Osteoporosis in aged males due to lower androgen levels and the effect of androgens on bone density have been subjects of consideration in recent studies.[14, 16] In males, androgens have been shown to be essential for skeletal maturation as well as maintaining the skeletal mass and bone density during mature stages.[17-19] Altogether, the effect of steroid sex hormones on bone turnover is inevitable.

There were studies designed to evaluate the effect of different bone turnover conditions induced by hormonal changes on orthodontic tooth movements and root resorption following orthodontic treatments. These conditions included hyperparathyroidism,[20] hyper and hypothyroidism,[21-22] lactating,[23] and ovariectomization.[2, 9, 24] The influence of castration before puberty was also investigated in skeletal maturation.[25-26]

Increased bone turnover in osteoporotic ovariectomized rats resulted in greater rates of OTM; however, few studies have demonstrated conflicting results on the OIRR rate in cases of estrogen and progesterone deficiencies.[2, 27] Therefore, the present study was designed to evaluate the effect of estrogen and progesterone deficiencies on OTM and OIRR of maxillary first molars 21 days after force application with coil springs in female castrated mature rats compared with healthy mature female samples. Moreover, as far as the authors are aware, there were no previous studies on the effects of androgen deficiencies concerning OTM and OIRR. Therefore, these issues were also evaluated and compared between healthy and testosterone-deficient mature male rats followed by castration 21 days after force application.

## Materials and Method

Twenty female and 20 male post-pubertal Wistar rats, aged 60 days, were used in this study. Ethical clearance of this study was obtained according to the guidelines of Animal Committee in Dental Research Center of Shahid Beheshti University of Medical Sciences. Ten female rats were randomly placed in the control group and the other ten were selected for ovariectomy as the experimental group. In the male samples, 10 rats were randomly assigned to orchiectomy and the rest were placed in the control group. All the rats were housed for 7 days in the same place with similar temperature and light conditions and fed with the same diet for adaptation with the environmental conditions. 

**Castration procedure**

The rats were weighed with a digital weighing scale and anesthetized with peritoneal injection of 10% ketamine at a dose of 50 mg/kg (Alfasan; Woerden, Holland) and 2% xylazine at a dose of 5 mg/kg (Alfasan; Woerden, Holland). Blood samples of all the rats were obtained from the retro-orbital venous plexus. Afterwards, bilateral orchiectomy (ORX) on experimental male rats and bilateral ovariectomy (OVX) on experimental female rats were performed. The samples in control groups underwent sham-operation with similar procedures except for the removal of ovaries and testes, with the purpose of attaining the same stress level as the experimental groups. After 4 weeks, blood samples were collected again from each rat. To confirm the success of castration procedure, the estrogen and progesterone levels in female rats and the testosterone level in male rats were measured by ELISA kits (Diagnostic Biochem Canada Inc.) before and after the procedure.

**Orthodontic appliance placement**

After confirmation of castration, the rats were sedated once again and a slot was shaped on the labial surface of the right maxillary incisor by a high-speed handpiece. A stainless steel closed-coil spring (0.008×0.030 inch) (Dentaurum; Ispringen, Germany) with the length of 8 mm was attached anteriorly to the right maxillary incisor and posteriorly to the first right molar with a steel ligature wire and the attachments were strengthened by adhesive resin (Transbond XT; 3M, Unitek). The appliance was set to induce a 60 g-force. The force applied was measured by a force gauge (Dentaurum; Ispringen, Germany). The appliance was not reactivated during the experience and was designed to induce a tipping movement in the first maxillary molar.

**OTM measurement**

Twenty-one days after force application, the rats were sacrificed by an overdose of ketamine hydrochloride. The distance between the distal surface of the first and mesial surface of the second right maxillary molar was measured by the same examiner with an electric caliper accurate to 0.001 mm (Tide Machine Tools Supply Co.; Shanghai, China). 

**OIRR and histomorphometric analysis**

The maxillary jaws were excised and after 7 days of decalcification, the first molars were embedded in paraffin blocks, and then serial 5-µm mesiodistal sections were cut. The central sections which showed the largest root surface were prepared by hematoxylin and eosin (H&E) staining. The mesial root was selected for evaluating the root resorption in this study because it is the largest root among the five roots of first maxillary molars and its alignment is approximately parallel to the applied force. Pictures were taken by a digital camera (Nikon E-8400; Tokyo, Japan) under a light microscope (Nikon Eclipse E-400; Tokyo, Japan) at 40x and 100x magnification. The mean of resorptive lacunae on the mesial surface of the mesial root was calculated in mm² by measuring the length and width of each resorptive lacuna with Adobe Photoshop 8 software (Adobe System; San Jose, CA) for histomorphometric analysis.

**Statistical analysis**

The descriptive values of root resorption and tooth movement were analyzed by SPSS 17.0 (SPSS Inc; Chicago). The normal distribution of values was determined by Kolmogorov-Smirnov test. The independent t-test was used to compare the values between the control and test groups. 

## Results

The hormone levels in the control groups at baseline and after 4 weeks were evaluated. The mean hormone levels in the experimental groups were measured before castration and 4 weeks after the operation. The descriptive mean values are presented in tables 1 and 2.

**Table 1:** The mean hormone levels in female groups

**Group**	**Hormone**	**Mean Value Before Operation**	**Mean Value 4 Weeks After Operation**	**Unit**
Control	Estrogen	196.9±61.65	94.57±10.5	pg/mL
	Progesterone	15.6±4.92	10.38±3.8	ng/mL
Experimental	Estrogen	102.31±12.44	67.63±11.88	pg/mL
	Progesterone	10.7±4.46	4.27±2.38	ng/mL

**Table 2:** The mean hormone levels in the male group

**Group**	**Hormone**	**Mean Value Before Operation**	**Mean Value 4 Weeks After Operation**	**Unit**
Control	Testosterone	5.3±2.68	4.53±2.34	ng/mL
Experimental	Testosterone	7.43±2.11	0.002±0.0063	ng/mL

The independent t-test showed significant differences in the mean progesterone and estrogen levels before and after ovariectomy (*p*= 0.001). The results of tooth movement and root resorption were compared with independent t-test and significant differences were detected between the control and experimental groups in male and female rats (*p*=-0.001). Tooth movement was significantly higher in the ovariectomy group and the rate of root resorption was greater in the control group.

Tooth movement was measured 0.70±0.07 mm in the experimental group and 0.54±0.06 mm in the control group. Root resorption in control group was 0.0081±0.0005 mm² and 0.0043±0.0006 mm² in the experimental group.

According to the results of independent t-test, the mean testosterone level in experimental male group was significantly different before and after castration, indicating the success of castration (*p*= 0.001).

In male groups, the castrated rats exhibited root resorption of 0.0041±0.0007 mm² which was lower than the control group with OIRR of 0.0079±0.0004 mm². Orthodontic tooth movement in castrated rats was higher (1.04±0.11 mm) than that in the other group (0.63±0.11 mm).

The mean values of tooth movement and root resorption followed by orthodontic forces are summarized in Table 3.

**Table 3:** The mean values of root resorption and tooth movement

	**OVX Group**	**Female Control Group**	**P Value**	**ORX Group**	**Male Control Group**	**P Value**	**Unit**
Tooth Movement	0.7±0.07	0.54±0.06	0.001	1.04±0.11	0.63±0.11	0.001	mm
Root Resorption	0.0043±0.0006	0.0081±0.0005	0.001	0.0041±0.0007	0.0079±0.0004	0.001	mm²

## Discussion

Increased demands for orthodontic treatment in older patients make it necessary to have a brief understanding of hormonal changes and their influence on the process and prognosis of treatment. The current study was performed to demonstrate the influence of sex hormones on orthodontic treatments. The results revealed that lack of estrogen and progesterone as well as testosterone increased the tooth movement and reduced the risk of orthodontic-induced root resorption.

Based on previous studies, OTM is accelerated in estrogen deficiencies. The current study also reached similar result as the mean value of OTM in OVX group was 0.7±0.07 mm compared with the control group with OTM of 0.54±0.06 mm.[2, 9, 28-29] In some studies the ovarian cycle and its relation with OTM have been investigated. Celebi *et al.* in an experimental study on cats reported a negative correlation between the estradiol level and OTM.[7] Another study on Wistar rats by Haruyama *et al.* showed OTM was about 33% greater in estrous level than pro-estrous level and suggested that OTM could be related to estrous cycle through its effect on bone resorption.[30] In this study, due to the decrease in both progesterone and estrogen levels after ovariectomy; it was not possible to evaluate the effect of progesterone on OTM exclusively. It has been assumed that progesterone had a specific role in maintaining skeleton integrity.[31-32] In some experimental studies, the effect of progesterone on orthodontic tooth movement has been investigated in pregnant animals. Ghajar *et al.* showed a lower OTM in pregnant rats than non-pregnant ones, although it was not statistically significant with lower osteoclasts on the tension side.[33]

It was previously demonstrated that testosterone affected the skeletal maturation and condylar growth while comparing the patterns in castrated and non-castrated neonatal rats.[19, 26] Previous studies on rat models reported the tooth movement to be divided into 3 phases.[2] In this study the samples were all sacrificed on day 21, while the third phase of tooth movement with an incremental line of increase should have been observed afterwards according to other studies. In future studies, the amount of tooth movement in days 1, 3, 7, 28 is suggested to be evaluated especially in testosterone deficiencies as studies have not investigated its influence on OTM previously.

As mentioned before, OIRR is a multifactorial complication of orthodontic treatments related to the patients’ conditions and treatment factors. The tooth root morphology and abnormalities, previous history of root resorption, genetics, type of malocclusion,[4] history of trauma and systemic conditions such as asthma, allergy,[34-35] drugs (Nabumetone) and hormone deficiencies altering bone remodeling like hypothyroidism[21-22]and secondary hyperparathyroidism due to calcium deficiency, serum calcium level[23, 36] and alveolar bone density[1] are factors influencing the susceptibility to OIRR. The magnitude, direction, and type of tooth displacement, duration of treatment and the continuous/interrupted force application and type of appliance are factors interfering with OIRR susceptibility.[1, 3]

In this study, the effects of sex steroid hormone levels on OIRR were investigated. The histomorphometric analysis of OIRR under light microscope showed a lower surface of OIRR in the OVX group than the control group. Sirisoontorn *et al.* showed an increase in OIRR in ovariectomized rats, which is not in agreement with the results yielded by this study.[2] In another study designed by Sirisoontorn *et al.*, the OIRR decreased to the limit of the control group by the use of zoledronic acid, a bisphosphonate in ovariectomized rats and the ovariectomized rats showed greater rates of OIRR.[28] The mentioned studies evaluated OIRR in distal and mesial roots of maxillary first molar; while in our study, only mesial roots were evaluated. Sirisoontorn *et al.* reported no statistically significant difference in root resorption between the control and OVX in the mesial root; however, the groups were significantly different concerning the distopalatal and distobuccal roots.[2] This difference could be due to the different angulation and morphology of the roots.

In ORX group, the OIRR was also decreased in comparison with the control group. To the best of authors’ knowledge, no studies have investigated the relation between testosterone deficiency and OIRR previously.

These differences in OIRR might be due to the imbalance in bone turnover. Previous studies demonstrated the effect of imbalanced bone turnover conditions due to other hormonal conditions on OIRR. Poumpros *et al.* claimed that higher bone turnover due to hyperthyroidism, induced by thyroxin application, resulted in lower root resorption[21] and Becks suggested that hypothyroidism is positively associated with OIRR.[37] As estrogen deficiencies are believed to increase bone turnover,[9] like in hyperthyroidism was condition, the results of this study were in accordance with what was found by Poumpros *et al.*[21] and lack of testosterone may have the same influence on bone turnover. However, another study by Verna *et al.* indicated no significant influence of bone turnover on OIRR.[27]

The steroid hormones such as estrogens, corticosteroids, androgens and progesterone and also related hormones (vitamin D, thyroid hormone, and the retinoids) act via structurally homologous nuclear receptors that form part of the steroid/thyroid receptor superfamily. Endocrine regulation occurs either by hormones acting at the cell membrane, for example the classical bone hormones, parathyroid hormone, and calcitonin, or by other hormones such as growth hormone and insulin. Evidence from a number of *in vitro*, *in vivo*, and clinical studies clearly indicates a role for steroid hormones in the regulation of normal bone development and the maintenance of intact bone.[38] Sex steroids also influence bone homeostasis. Estrogen suppresses bone resorption by reducing osteoclast numbers.[39] Testosterone reduces bone resorption in males, promotes bone formation in males and females, and can be converted to estrogen to inhibit bone resorption. Still much remains to be learned about estrogen mediated bone resorption.[39]

Estrogen physiological fluctuations can cause physiological fluctuations in the serum markers of bone turnover; moreover, by binding to its receptors in periodontal tissue, it regulates the remodeling of alveolar bones, promotes bone formation, and inhibits bone resorption.[40] Furthermore, increasing the levels of estrogen in periodontal tissues enhances the secretion of osteocalcin (OCN); hence, inhibits bone resorption and increases bone formation.[41] Estrogen inhibits bone resorption directly by regulating specific gene activities and by inducing genes that trigger apoptosis, consequently, reducing the number of osteoclasts.[42]

Yamashiro and Takano-Yamamoto showed that estrogen withdrawal, which is important in the pathogenesis of post-menopausal osteoporosis, accelerated bone metabolism with a negative calcium balance. They also concluded that estrogen deficiency caused significantly rapid orthodontic tooth movement, and that the acceleration of tooth movement could be due to the further activation of alveolar bone turnover.[24]

Bartzela *et al.* performed a systematic literature review on the effects of medications on the rate of orthodontic tooth movement and concluded that estrogen supplements would probably reduce tooth movement, although no direct evidence was available.[43]

Testosterone deficiency is a known risk factor for osteopenia and osteoporosis in older men. Less is known about the impact of testosterone deficiency on bone mineral density in younger men. The study of Kacker *et al.* reported that more than one third of men younger than 50 years with testosterone deficiency and infertility or sexual dysfunction had decreased bone mineral density and testosterone treatment increased the bone mineral density.[44]

These controversies show the necessity of further investigations about the influence of different bone turnover conditions on OIRR. The different methods used in these studies may have been the logic for all these controversies. More studies on the effect of sex steroid hormones on OPG/RANK/RANKL system and expression of cytokines in tension and pressure sides may be the clue to solve the problem. Further studies on the effect of testosterone deficiencies in mature cases are also suggested. The risks of complications such as root resorption and unpredictable tooth movement associated with sex hormone reductions due to aging in both male and female patients must be fully understood to plan an accurate treatment in older patients. 

In addition, consumptions of hormonal drugs may have similar effects that should be considered in orthodontic patients. 

## Conclusion

In female samples, the OVX group showed an increase in OTM with a lower rate of OIRR and in male groups, the ORX group showed an increase in OTM with a lower rate of OIRR (*p*= 0.001). Alterations in steroid sex hormones can influence the amount of OTM and RR. Controversial results of previous studies necessitate further investigations to evaluate the influence of different bone turnover conditions that are related to sex steroid hormones and have an impact on OIRR.
